# Right Hepatic Artery Syndrome With Mirizzi Syndrome: A Case Report

**DOI:** 10.7759/cureus.34559

**Published:** 2023-02-02

**Authors:** Ibrahim Hazazi, Hassan Alyami, Tumadher Alowairdhi, Leena Almaghrabi, Abdulaziz A ALQahtani

**Affiliations:** 1 General Surgery, King Fahad Military Medical Complex, Dammam, SAU; 2 General Surgery, Security Forces Hospital, Dammam, SAU

**Keywords:** cholecystectomy, mirizzi syndrome, hepatic artery syndrome, hepatic, bile

## Abstract

Anatomical diversity is rather typical in the biliary region. However, it has only sometimes been documented that the arteries of a hepatobiliary origin compressed the extrahepatic bile duct. Biliary obstruction may be caused by a myriad of benign and malignant diseases. Right hepatic artery syndrome (RHAS) is described as the consequence of right hepatic artery compression of the extrahepatic bile duct. We report a case of a 22-year-old male who presented with a complaint of abdominal pain and was later admitted as a case of acute calculous cholecystitis with obstructive jaundice. Abdominal ultrasound showed a picture of the so-called Mirizzi. However, A magnetic resonance cholangiopancreatography showed a picture of RHAS, so the patient needed endoscopic retrograde cholangiopancreatography to decompress the biliary system which was later performed successfully followed by cholecystectomy. The diagnosis of RHAS is well established in the literature, and it depends on the facility of the institute, cholecystectomy versus hepaticojejunostomy or endoscopic treatment alone are the management options that have been utilized to manage such cases.

## Introduction

The liver has a dual blood supply, receiving blood from the portal vein and the hepatic artery. The right lobe of the liver is supplied by a typical or normal right hepatic artery, which emerges from the proper hepatic artery. The origin and course of the right hepatic artery are prone to anatomical variations [1]. The hepatic portal is defined anatomically as a bundle of luminal structures such as the bile duct, portal vein, and hepatic artery. Other organs, like the esophagus, duodenum, and ureter, have vessels that press on the neighboring organ and obstruct the flow of its contents. However, the right hepatic artery compressing the bile ducts has only occasionally been documented. The first instance of hepatolithiasis and jaundice caused by right hepatic artery compression on the common hepatic duct was documented by Tsuchiya et al. in 1984. Since then, this condition is referred to as right hepatic artery syndrome (RHAS) [2,3].

A relatively rare complication of acute cholecystitis is the development of a hepatic artery pseudoaneurysm; nevertheless, a pseudoaneurysm causing gallbladder neck compression and intrahepatic duct dilation cause a presentation similar to Mirizzi syndrome which is mostly unreported [4]. Mirizzi syndrome is a challenging condition to identify and treat, and biliary surgeons aware of the risk for their patients face additional difficulty. The disease can mimic gallbladder cancer but also manifests as a precancerous condition, making diagnosis very challenging. In addition, cholecystectomy is a particularly risky procedure due to the considerable rise in the chance of intraoperative biliary damage. Due to the varied clinical presentation, a standard treatment for Mirizzi syndrome has not yet been established. Planning for surgical intervention should come after a thorough evaluation of the local environment and anatomy [5]. We present a case of a 22-year-old male who was admitted with a case of acute calculous cholecystitis with obstructive jaundice but was later diagnosed with Mirizzi syndrome.

## Case presentation

This is a case of a 22-year-old male not known to have a chronic medical illness and no significant medical or allergic history except surgical background of sleeve gastrectomy two years ago where he lost around 65 kg. The patient had a positive paternal history of pancreatic cancer. He complained of abdominal pain which lasted for 10 days on and off, associated with darkened urine, constipation, and anorexia. There is no history of nausea, vomiting, fever, or itching. Generally, the patient was not distressed but was deeply jaundiced. The patient was admitted with a case of acute calculous cholecystitis with obstructive jaundice. Upon examination, the patient was vitally stable with a blood pressure of 123/87 mmHg, a temperature of 36.8°C, a heart rate of 53 per minute, a respiratory rate of 20 per minute, and an oxygen saturation of 100%. Chest examination revealed equal bilateral air entry. Furthermore, his abdominal examination revealed right upper quadrant tenderness and a positive Murphy sign. A previous laparoscopic surgical scar was present. Laboratory investigations of the patient are depicted in Table 1.

**Table 1:** Laboratory investigation profile

Investigation	Value	Normal value Reference range
Hemoglobin	14.9 g/dL	13.8 to 17.2g/dL
White blood cell	3.38 x10113/uL	4,500 to 11,000per microliter (4.5 to 11.0 × 10^9^/L)
Platelets	287 x10113/uL	150,000 to 450,000 microliter
Albumin	32.3	34 to 54 g/L
Alkaline phosphatase	89.7	44 to 147 IU/L
Direct bilirubin	111.08 µmol/L	less than 5.1 µmol/L
Total bilirubin	129.3 µmol/L	1.71 to 20.5 µmol/L
Aspartate aminotransferase	57.3 U/L	8 to 33 U/L
Alanine transferase	152.7 U/L	4 to 36 U/L
Creatinine	42 µmol/L	65.4 to 119.3 micromoles/L
Blood urea nitrogen	1.4 mmol/L	2.1 to 8.5 mmol/L
Sodium	134.6 mmol/L	136 and 145 mmol/L
Potassium	4.1 mmol/L	3.6 to 5.2 mmol/L

**Figure 1:**
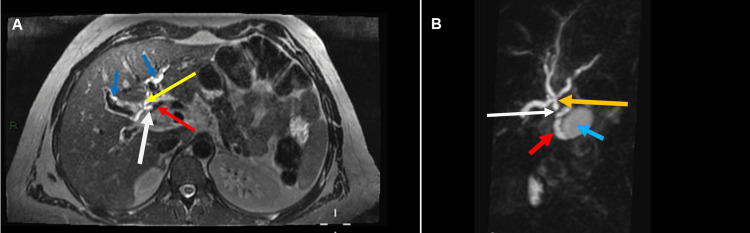
(A) Axial T2 image showing curvilinear signal flow void (red arrow) from the right hepatic branch crossing the common hepatic duct (white arrow) which shows abrupt cut-off at the point of vessel crossing and mild dilatation along with dilated major hepatic ducts exhibiting trifurcation variation of common hepatic duct division (yellow arrow) as well as prominent intrahepatic biliary channels (blue arrows). (B) MRCP Coronal 3D reconstructed image shows trifurcation variation of common hepatic duct division (yellow arrow) and abrupt cut-off at the proximal CHD by crossing right hepatic branch, resultant upstream intrahepatic biliary dilatation and downstream collapsed CBD (No CBD luminal signals). Ovoid hyperintensity (blue arrow) represents gallbladder with adjacent curvilinear hyperintensity denoting cystic duct (red arrow).

An abdominal ultrasound showed that the liver was of normal size. However, coarse parenchymal echotexture was noted, which could be correlated with a hepatitis profile. The gallbladder showed multiple mobile tiny gallbladder stones, with minimal thickening of the gallbladder wall reaching up to 5 mm; however, no pericholecystic free fluid was seen. The common bile duct diameter appeared prominent, measuring 5 mm, with minimal proximal intrahepatic biliary duct dilatation. Further evaluation by magnetic resonance cholangiopancreatography was recommended. No common bile duct stone was detected at the visualized part of the proximal common bile duct (Figures 1A, 1B).

**Figure 2:**
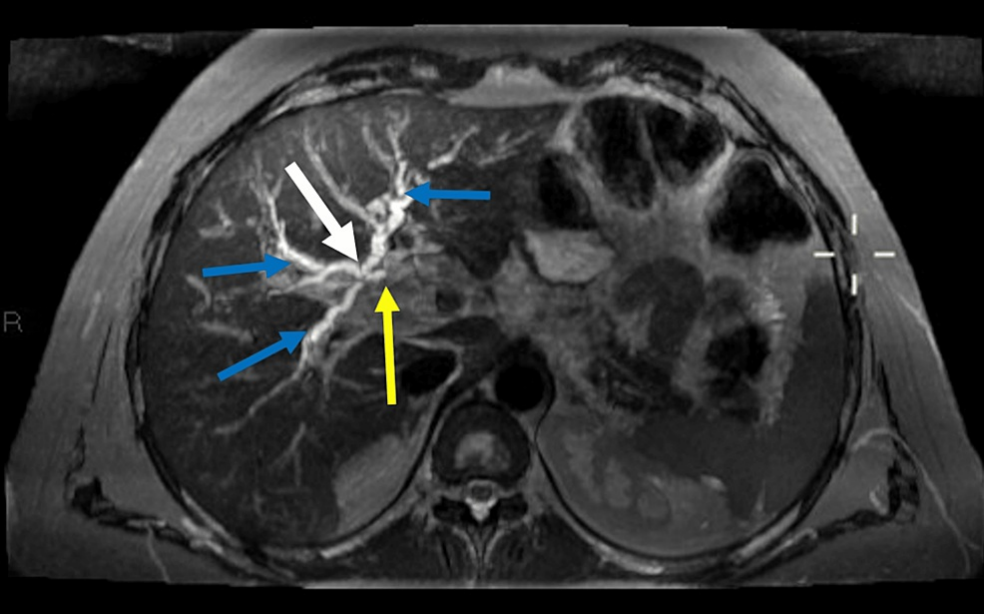
Axial T2 FAT suppressed MIP image showing trifurcation variant of CHD division (white arrow), mild intrahepatic biliary dilatation (blue arrows), mildly dilated proximal common hepatic duct with abrupt cut off (Yellow arrow).

MRCP showed that the gallbladder was distended, containing multiple small calculi along with diffuse gallbladder wall thickening measuring up to 8 mm with a streak of pericholecystic free fluid extending into the subhepatic space in keeping with acute calcular cholecystitis. A small cystic duct stone was seen causing the obstruction. There was a trifurcation anomaly of the intrahepatic biliary tree, intrahepatic biliary channels showing dilatation, and a slightly beaded appearance, which could be related to cholangitis (Figure 2).

**Figure 3:**
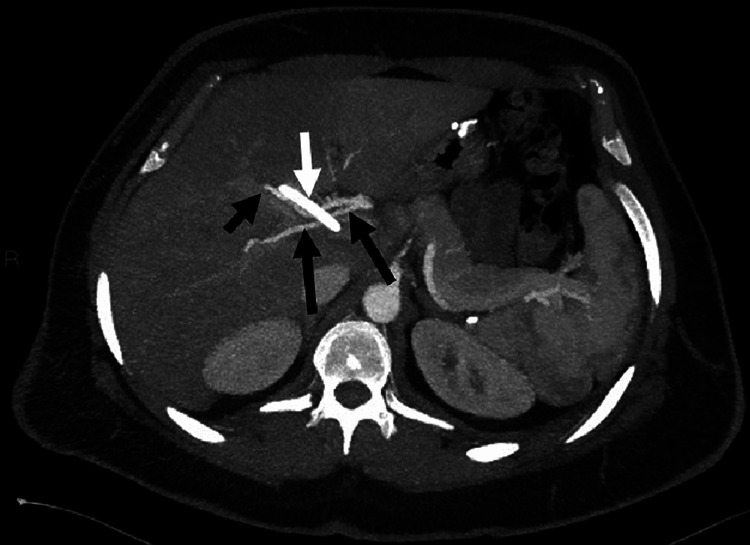
Axial contrast-enhanced CT arterial phase MIP image shows crossing right hepatic artery branches (black arrows) against the stented common hepatic duct (white arrow). Regression of intrahepatic biliary dilatation post stenting.

Furthermore, there is an abrupt cut-off at the level of the proximal common hepatic duct beyond which the distal common hepatic duct and proximal common bile duct were not visualized/collapsed. A prominent vessel was seen crossing at the site of the cut-off which could be responsible for the focal narrowing along the retrograde intrahepatic dilatation as well as the focal structuring related to the cholangitis (Figure 3). Moreover, the distal common bile duct was visualized and is of normal caliber. The liver was mildly enlarged measuring 17.5 cm with a small volume lymph node measuring 10 x 7 mm at the porta hepatis. There were no remarkable pancreas or pancreatic duct changes despite having the small splenunculus at the splenic hilum prominent. The rest of the abdominal viscera appeared grossly unremarkable.

Upon admission, the patient was kept NPO with intravenous fluid and analgesia along with the antibiotic for ultrasound and magnetic resonance cholangiopancreatography. The gastroenterology team was involved from the time of admission. The findings of the ultrasound and magnetic resonance cholangiopancreatography showed a picture of Mirizzi syndrome and the possible need for endoscopic retrograde cholangiopancreatography to decompress the biliary system which was performed in another hospital where the patient was accepted. The procedure was done without any complications and was successful as the patient’s symptoms improved.

## Discussion

Jaundice or the development of gallstones has been attributed to anatomical variations of the hepatic artery. Bove et al. reported three cases of intrahepatic stones caused by the right hepatic artery compressing on the proximal common bile duct. In order to remove intrahepatic stones, the three patients had endoscopic retrograde cholangiopancreatography and biliary sphincterotomy. After the stones were removed, cholangiography revealed common bile duct compression immediately below the main hepatic confluence, which was later proven to be owing to right hepatic artery compression on MRI [6]. Contradictory to this our patient showed multiple stones in the gallbladder, acute calcular cholecystitis, and a small cystic duct stone which was responsible for the obstruction although RHAS was likewise present. Similar to our case Anderson et al. reported a case of a 54-year-old man who also presented with obstructive jaundice although the age of our patient differed. Angiography revealed a pseudoaneurysm of an embolized right hepatic artery branch during an incident of hemomobilia. During surgery, it was discovered that a gallstone that causes Mirizzi type II syndrome is what is causing the biliary obstruction, along with a necrotic inflammatory mass and hematoma that was expanding into the liver. The obstruction-causing stones were removed, the mass was debrided and drained, and a t-tube was used to empty the bile duct. The patient recovered completely [7].

Mirizzi syndrome and other chronic complications of symptomatic gallstone diseases are uncommon. When detected during a laparoscopic cholecystectomy, this condition's relevance and consequences are related to its associated and potentially serious surgical sequelae, such as bile duct injury, and its current therapy. A pressure ulcer caused by an impacted gallstone at the gallbladder infundibulum, followed by an inflammatory response that initially blocked the bile duct externally before eroding into the duct and developing into a cholecystocholedochal or cholecystohepatic fistula, has been used to explain the pathophysiological process leading to the subtypes of Mirizzi syndrome [8]. A prolonged cystic duct that runs parallel to the bile duct and the low insertion of the cystic duct into the common bile duct have both been proposed as predisposing factors for the development of Mirizzi syndrome. Gallstones that are impacted repeatedly or continuously may result in recurrent acute cholecystitis attacks and cause the gallbladder to become initially distended with thick inflammatory walls before contracting and becoming atrophic with thicker fibrotic walls [9]. The magnetic resonance cholangiopancreatography findings of the patient also depicted a distended gall bladder and cholangitis was also present.

Cui et al. described that the diagnostic sensitivity of Mirizzi syndrome may be increased by combining choledochoscope surgery with ultrasound, magnetic resonance cholangiopancreatography, and endoscopic retrograde cholangiopancreatography. It is effective to confirm Mirizzi syndrome during surgery using an intraoperative choledochoscope. The current gold standard for treating Mirizzi syndrome patients is open surgery. Patients should be carefully chosen for laparoscopic surgery, which should be restricted to those with type I Mirizzi syndrome [10]. For the diagnosis of Mirizzi syndrome, endoscopic retrograde cholangiopancreatography is the gold standard. It defines ductal anomalies, such as fistula, and the source, degree, and extent of the biliary blockage. Additionally, endoscopic retrograde cholangiopancreatography provides a range of treatment alternatives, including biliary stent implantation and stone removal. Similar information can be obtained through endoscopic retrograde cholangiopancreatography and percutaneous cholangiogram; however, endoscopic retrograde cholangiopancreatography has the advantage of being able to spot low-lying cystic ducts that percutaneous cholangiogram might miss. High-resolution pictures of the biliary tract and surrounding tissues can be obtained using wire-guided intraductal ultrasonography. Arterial impulses cause of the blood flow could falsely give a beaded hepatic configuration and might lead to a presumption of other differential diagnosis like cholangiocarcinoma or cholangitis. POCS with spyglass DS system has been utilized successfully to diagnose hepatic artery syndrome which was not available in our facility instead MRI was used and gave a wide range of possibilities which we needed to rule out before moving to the definitive management [2]. Stojanovic et al. reported a case of biliary obstruction mimicking the klatskin tumor based on a CT scan. Intraoperatively, a cholangiogram was done, and the diagnosis of RHAS was established the patient underwent cholecystectomy with careful delineation of the biliary anatomy the patient was discharged in good condition [11]. In our case, the patient had a combined Mirrizi and RHAS the patient underwent laparoscopic cholecystectomy with meticulous dissection of the biliary tree. The patient had a smooth post-operative course and was discharged later in a good health. The main mode of treatment is surgery [11]. The current gold standard for treating Mirizzi syndrome patients is open surgery [12].

## Conclusions

Mirizzi syndrome accompanied by RHAS is a rare presentation. Abdominal ultrasound might not be the optimal tool for diagnosis as it might show signs of inflammatory hepatitis which can be misguiding in young adults. The magnetic resonance imaging is the golden standard to visualize the biliary tree. Surgery is the treatment choice in such cases as cholecystectomy was performed and the patient improved afterward. Further research focusing on Mirizzi syndrome can be beneficial in understanding the clinical characteristics of the condition.
